# Immunological responses of septic rats to combination therapy with thymosin α1 and vitamin C

**DOI:** 10.1515/biol-2022-0551

**Published:** 2023-02-07

**Authors:** Daquan Zhang, Lu Wang, Zhigao Wang, Xiaohui Shi, Wen Tang, Long Jiang, Ba Yin Cha Han Bo Ran Yi, Xinwei Lv, Congyu Hu, Dong Xiao

**Affiliations:** Department of Critical Care Medicine, People’s Hospital of Xinjiang Uygur Autonomous Region, Urumqi 830001, Xinjiang, China

**Keywords:** sepsis, thymosin α1, vitamin C, immune response, inflammatory response

## Abstract

This study investigated the effect of combined thymosin α1 and vitamin C (Tα1 + VitC) on the immunological responses of septic rats. Five groups were designed. The septic model was established by the cecal ligation puncture (CLP) method. The sham group did not undergo CLP, the model group was given normal saline solution, the Tα1 group was given Tα1 (200 µg/kg), the VitC group was given VitC (200 mg/kg), and the Tα1 + VitC group was given Tα1 + VitC. Specimens for immunological analyses were collected at 6, 12, 24, and 48 h posttreatment in each group except for the sham group (only at 48 h). CD4 + CD25 + T cells in the peripheral blood and dendritic cell (DC) proportions in the spleen were analyzed by flow cytometry. Tumor necrosis factor α (TNF-α), interleukin 6 (IL-6), transforming growth factor-β (TGF-ß1), and nuclear factor kappa-B (NF-κB) were measured by ELISA. CD4 + CD25 + T cells and OX62 + DCs levels significantly increased in the model group and decreased in the Tα1 and/or VitC treatment groups. Similarly, the levels of TNF-α, IL-6, TGF-ß1, and NF-κB significantly increased in the model group and decreased in the Tα1, VitC, and Tα1 + VitC groups, indicating that combined Tα1 and VitC therapy may help regulate the immunological state of patients with sepsis, thereby improving prognosis.

## 1 Introduction

Sepsis is a series of immune response dysregulation syndromes that occur after infection with pathogenic microorganisms, and sepsis-related infectious shock and multiorgan dysfunction syndrome are the main causes of patient death [1,2,3,4]. The high incidence and lethality of sepsis is a serious threat to human health. Statistically, the annual incidence of sepsis is 0.149–0.24%, while the annual mortality rate is 28–50% [5,6]. Each case of sepsis is costly and imposes a huge economic burden on society.

Sepsis has long been thought to be divided into two distinct phases: the proinflammatory response phase and the anti-inflammatory response phase. The proinflammatory phase typically occurs first. The intrinsic immune system releases systemic inflammatory factors that fight infection; the adaptive immune system is simultaneously recruited and increases the intensity of the immune response. The anti-inflammatory response phase refers to systemic inflammatory response syndrome (SIRS), which begins immediately after the proinflammatory response phase and is generally considered an irresponsive phase of infection [7]. When pathogens infect the body, the pathogen antigens are recognized by receptors on the surface of inflammatory effector cells. Nuclear factor kappa-B (NF-κB) is activated through an antigen-antibody response and enzyme activation, promoting the generation and release of various proinflammatory factors and inflammatory mediators, such as interleukin 6 (IL-6), tumor necrosis factor α (TNF-α), and transforming growth factor-β (TGF-β). NF-κB was first discovered by David Baltimore and is a transcription factor that can aggravate the tissue inflammatory response. NF-κB activation or phosphorylation can transactivate cyclooxygenase-2 or allograft inflammatory factor-1 expressed in inflammatory or malignant tumors, and NF-κB can activate multiple inflammatory factors, such as TNF-α, IL-6, IL-8, and matrix metallopeptidase-9 [26]. IL-6 is a cytokine involved in multiple immune responses and is associated with several diseases [25]. TNF-α is a potent proinflammatory cytokine with deleterious effects in several autoimmune diseases that also mediates paradoxical anti-inflammatory and immunomodulatory effects. TGF-β belongs to a newly identified TGF-β superfamily that regulates cell growth and differentiation. In recent years, TGF-β has been shown to play important regulatory roles in cell growth, differentiation, and immune function. TGF-β inhibits the proliferation of immunologically active cells and the differentiation of lymphocytes and suppresses cytokine production. TGF-β can synergistically activate mouse thymic MHC nonrestricted killer cells via IL-2 and IL-6. TGF-β can also inhibit the production of interferon gamma (IFN-γ) and TNF-α in peripheral blood mononuclear cells. Numerous anti-inflammatory immunomodulatory treatments have been used to combat the proinflammatory phase of sepsis; however, these immunomodulatory agents (TNF-α, IL-1β, toll-like receptor-4, etc.) have failed in clinical trials [8,9,10]. This failure has led to the recognition that the pathogenesis of sepsis is not simply the result of an excessive proinflammatory response to infection, suggesting that the pathophysiological process of sepsis is a more complex condition.

Data suggest that the proinflammatory and anti-inflammatory phases occur simultaneously during the host immune response to severe trauma or sepsis [11]. The magnitude of the initial proinflammatory phase appears depending on many factors, including the virulence of the pathogen, pathogen load, and the patient’s underlying disease state. While the accumulation of proinflammatory factors in patients is associated with early morbidity and mortality, the number of chronic sepsis-related deaths appears clearly associated with immunosuppression and immune dysfunction. In fact, most sepsis-related deaths occur after 3 days, and some occur several weeks after the onset of disease [10]. Patients with sepsis pass through an early proinflammatory phase followed by a prolonged period of immunosuppression and immune paralysis. Numerous studies have led to a deeper and broader understanding of sepsis and the gradual recognition that its pathogenesis is complex. There is an excessive inflammatory response, immunosuppression, a second strike, oxidative stress, apoptosis, and coagulation disorders caused by the invasion of pathogenic microorganisms that participate in organ damage in septic patients [12,13]. Although many basic and clinical studies have been conducted on the pathogenesis and treatment of sepsis, no breakthroughs have been made. Immune disorders manifest as a disruption of the balance between excessive inflammatory responses and immunosuppression, which occur simultaneously, and the dynamic balance between excessive inflammatory responses and immunosuppression in the organism is restored through immunomodulation. Research reports on achieving immune regulation and restoring balance in sepsis and the related mechanisms are gradually increasing.

Thymidine α1 (Tα1) can have antimicrobial effects by increasing the release of IL-12, IL-2, IFN-α, and IFN-γ, and it also increases IL-10 secretion and the ratio of regulatory T cells (Tregs), controlling inflammatory responses [14,15]. Tα1 is widely used to regulate immune disorders in sepsis and is a new direction for sepsis treatment. Vitamin C (VitC) has anti-inflammatory and immunomodulatory effects on sepsis, and Fisher et al. [16] showed that VitC significantly attenuated the expression of proinflammatory chemokines and NF-kB activity in septic mice and prolonged their survival time. In addition, high mobility group protein B1 (HMGBl) triggers the inflammatory response by increasing the release of inflammatory factors (such as TNF-α, IL-1, IL-6, IL-8, and macrophage inflammatory protein 1B), and changes in HMGBl levels are significantly correlated with the prognosis of patients with sepsis [17,18]. High doses of VitC induce the expression of heme oxygenase-1, which reduces the release of HMGBl, attenuates the inflammatory response, and significantly increases the survival rate of septic mice [19]. VitC improves brain function and increases the survival rate of septic rats [20].

The mechanism of sepsis is complex and relies on a single drug with limited therapeutic efficacy, and outcomes may be improved by a combination of drugs. VitC combined with hydrocortisone and vitamin B1 has been tested clinically in the early stages of sepsis [21,22], and these agents can reduce the inflammatory response and decrease morbidity and mortality rates. Our group showed that combining Tα1 and hydrocortisone could improve immune function, regulate the inflammatory response, and increase the survival rate of mice with endotoxin-induced sepsis. There have been many studies on the effects of VitC on septic mice, but few have reported the immune response to thymidine α1 combined with VitC.

Based on the aforementioned information, this study investigated changes in the immune response in septic rats treated with Tα1 and VitC alone or in combination, analyzed the effect on the immunity of septic rats, and provided a theoretical basis for the study of immune regulation therapy in septic rats. The results of this study might shed new light on the treatment of sepsis and deepen the understanding of this condition.

## 2 Materials and methods

### 2.1 Animal modeling and groupings

A total of 85 male Sprague Dawley (SD) rats (weighing 220 ± 20 g) were provided by the Experimental Animal Center of the First Teaching Hospital of Xinjiang Medical University. The septic rat model was established by the cecal ligation puncture (CLP) method. The rats were randomly divided into the sham, sepsis model, Tα1, VitC, and Tα1 + VitC groups. (1) In the sham group (*n* = 5), after the rats were disinfected, a 1.5 cm-long longitudinal incision was made in the main midline of the lower abdomen, which was stratified into the abdominal cavity, completely exposing the cecum, and the mesenteric membrane was separated. The abdominal wall incision was stitched layer by layer. After the surgery, an equal amount of normal saline was injected into the abdomen. (2) The sepsis model group (*n* = 20) was established by using the CLP method. After the surgery, an equal amount of normal saline was injected into the abdomen. (3) In the Tα1 group (*n* = 20), the rat sepsis model was established by CLP, and 200 μg/kg Tα1 was dissolved in saline and injected into the abdomen. (4) In the VitC group (*n* = 20), the rat sepsis model was established by CLP, and 200 mg/kg VitC was dissolved in saline and injected into the abdomen. (5) In the Tα1 + VitC group (*n* = 20), the rat sepsis model was established by CLP, 200 μg/kg Tα1 was dissolved in saline and injected into the abdomen, and 200 mg/kg VitC was dissolved in saline and injected into the abdomen.

All groups except for the sham group were further divided into four groups, with five animals per group, according to the time points (6, 12, 24, and 48 h after treatment) for sample collection.


**Ethical approval:** The research related to animal use has been complied with all the relevant national regulations and institutional policies for the care and use of animals and has been approved by the Institute of Animal Ethics of Xinjiang Medical University (SYXK 2018-0001).

### 2.2 Sample collection

All rats in each group survived until the designated experimental time points.

At the designated experimental time point, the rats in the model, Tα1, VitC, and Tα1 + VitC groups were weighed and anesthetized with 5% chloral hydrate at 8 ml/kg by intraperitoneal administration, and the limbs were immobilized and photographed. At 48 h, the same procedure was performed on the rats in the sham group.

Blood was collected from the heart. The blood sampling needle was inserted into the left chest where the heartbeat was most obvious, and the anticoagulant tube/nonanticoagulation tube was connected. The needle was inserted slowly, and the tube was clamped when full. One anticoagulant tube was collected per rat. The anticoagulant tube was shaken gently and immediately refrigerated at 4°C. A nonanticoagulation tube was used to facilitate the separation of clear serum from blood, minimizing shaking as much as possible.

Then, the rat abdomen was opened to expose the spleen under the stomach and separated into two parts with five samples taken from each part, for a total of ten splenic tissue samples. Six samples were fixed in 4% formaldehyde and stored in 5–10 ml tubes at room temperature. The other four samples were frozen at −80℃ for grouping and labeling, and testing was performed 1–2 days later. Three samples were stained with hematoxylin-eosin (HE), enzyme-linked immunosorbent assay (ELISA) was performed on three samples, and the remaining four samples were analyzed by flow cytometry.

### 2.3 Flow cytometry

Flow cytometry was performed in rigorous accordance with the instructions of the kit (Solarbio, Beijing, China; article no., P8630), and five repetitions were performed for each group.

The rat blood samples were centrifuged to prepare a cell suspension, and 50 μl of direct-labeled mouse anti-CD4/FITC (Bioss, bsm-33076M-FITC) and direct-labeled Rb IL2RA/CD25/PE (BioLegend, 303504) antibodies in PBS were added, mixed well, and incubated at 4°C for 30 min. Then, 1 ml of PBS was added to wash the samples, which were centrifuged at 1,000 rpm for 5 min. The supernatant was discarded and 200 μl of PBS was added to resuspend the cells, which were then placed in a specific flow cytometer for analysis. FlowJo software was used for image processing, and GraphPad Prism 7.00 software was used for analysis and graph construction.

The rat spleen was ground until no obvious tissue clumps were seen, and the obtained suspension was passed through a 200-mesh filter screen. The culture dish was rinsed with PBS to collect the remaining cells and wash the screen, and the filtrate was placed in a centrifuge tube. The tube was centrifuged at 1,500 rpm for 8 min at 4°C, and the supernatant was discarded. Then, 1 ml of erythrocyte lysis buffer (ten times the cell volume ratio) was added to the centrifuge tube, and the sample was mixed thoroughly. After standing at room temperature for 5 min, 8 ml of precooled PBS was added, and the mixture was centrifuged at 1,500 rpm at 4°C for 8 min. The supernatant was discarded, an appropriate amount of precooled PBS was added, and the suspension was mixed well. A small volume of the suspension was taken for proper dilution, and the number of cells was adjusted to approximately 500,000 per tube. Then, 1 ml of PBS was used to wash the sample, which was centrifuged at 1,000 rpm for 5 min at 4°C, and the supernatant was discarded. Next 50 μl of antibody-containing PBS was added, and the sample was mixed well and incubated at 4°C for 30 min. The samples were washed with 1 ml of PBS and centrifuged at 1,000 rpm for 5 min, the supernatant was discarded, 50 μl of staining solution containing secondary antibody was added to resuspend the cells, and the samples were incubated at 4°C for 30 min. The samples were washed with 1 ml of PBS and centrifuged at 1,000 rpm for 5 min, and the cell supernatant was discarded. The cells were resuspended in 200 μl of PBS, placed in a specific test tube, and analyzed by a flow cytometer. FlowJo software was used for image processing, and GraphPad Prism 7.00 software was used for analysis and graph construction.

### 2.4 ELISA

Serum samples were collected at 48 h by centrifugation. The TNF-α, IL-6, TGF-β1, and NF-κB levels in the serum samples were measured with an ELISA kit (Shanghai Enzyme-linked Biotechnology, Shanghai, China) according to the manufacturer’s instructions.

Spleen tissue was ground by adding prechilled PBS. The TNF-α, IL-6, and TGF-β levels in the spleen samples were measured with an ELISA kit (Shanghai Enzyme-linked Biotechnology, China) according to the manufacturer’s instructions.

### 2.5 HE staining

The rat spleen tissues were soaked in 10% formaldehyde for 7 days, embedded in paraffin, sliced, and deparaffinized in a xylene solution. After full hydration, 100 µl of hematoxylin staining solution was added dropwise for 10 min, and then the sections were differentiated with 1% hydrochloric acid in ethanol. A weakly basic blue-stimulating solution was added to the tissue section to stain the nuclei blue. Eosin staining solution was added, and after 3 min, the tissue sections were dehydrated by a gradient. The dehydrated tissue sections were soaked in xylene twice for 4 min each, and then the tissue sections were dried and sealed with neutral gum. Images were taken with an upright microscope with a 100× field of view.

### 2.6 Statistical analysis

The data are expressed as the mean value ± standard deviation (SD). Statistical analysis was performed using SPSS 22.0 software. ANOVA was used to analyze the pairwise comparison after Bartlett’s homogeneity of variance test, and differences between the groups were compared by the least significant difference (LSD)-t method. *P* < 0.05 was considered statistically significant.

## 3 Results

### 3.1 Flow cytometry

The proportions of CD4 + CD25 + double-positive T cells in peripheral blood and OX62 + dendritic cells (DCs) in the spleen were counted by flow cytometry to investigate the effect of Tα1 and/or VitC treatment on the immune response in septic rats.

**Figure 1:**
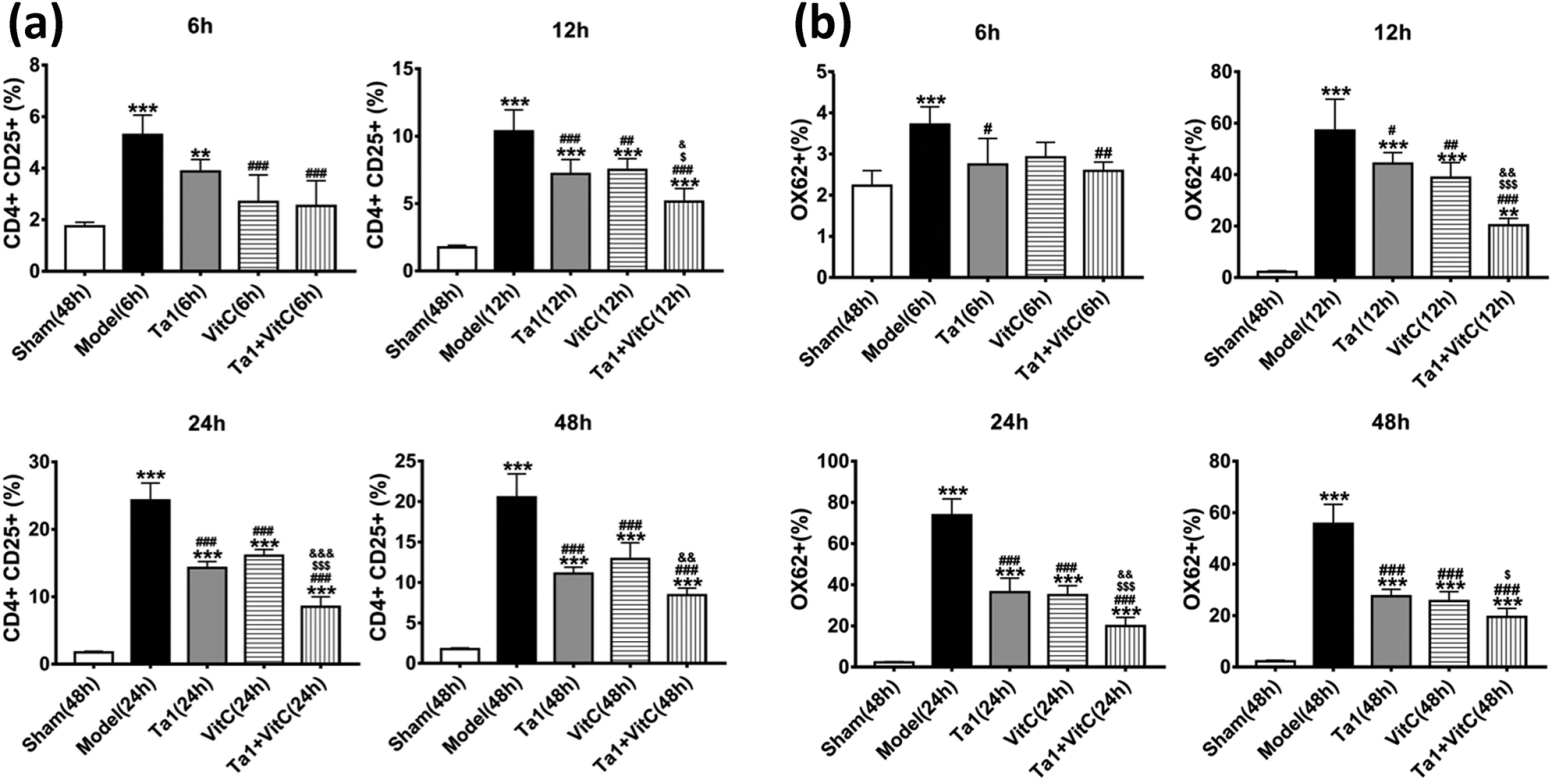
Comparison of the flow cytometry results among different groups. (a) CD4 + CD25 + T cells in serum. (b) OX62 + DCs in the spleen. Note: **P < 0.01 and ***P < 0.001 vs the sham group; #*P* < 0.05, ##*P* < 0.01, and ###*P* < 0.001 vs the sepsis model group; $*P* < 0.05 and $$$*P* < 0.001 vs the Tα1 group; &&*P* < 0.01 vs the VitC group.

**Figure A1:**
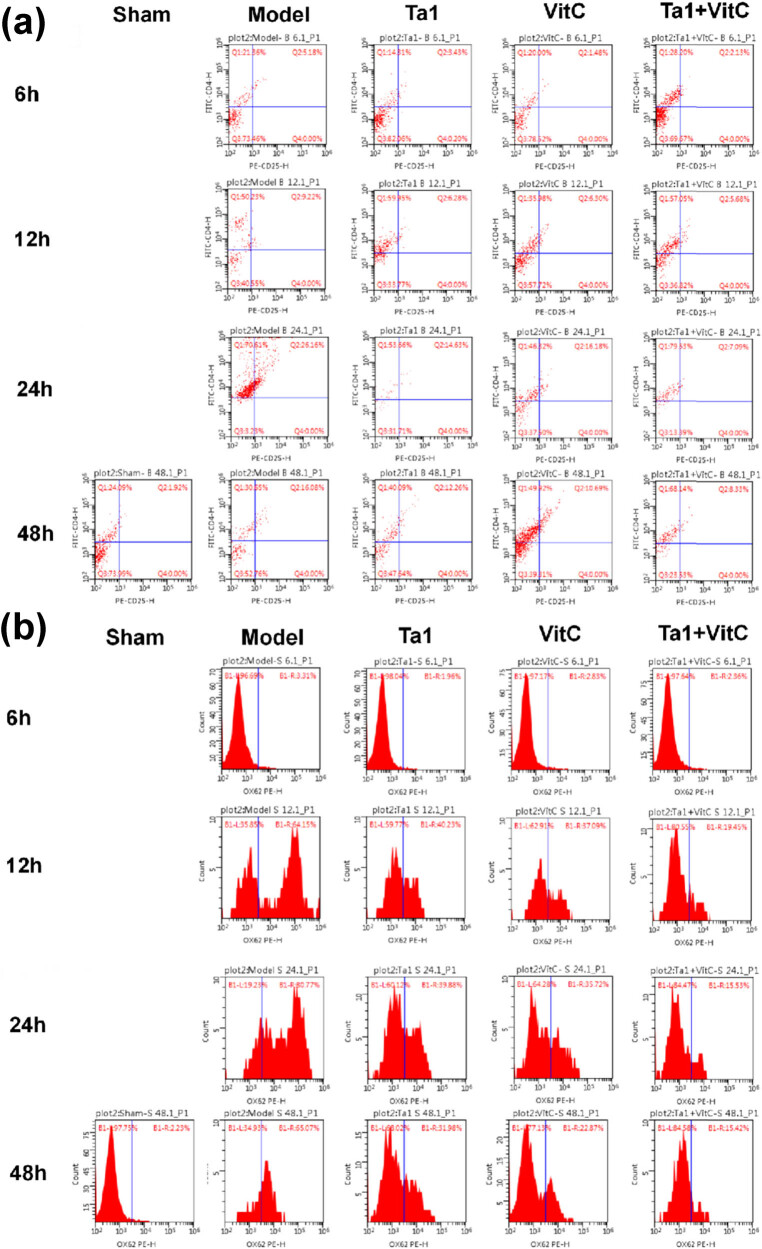
Flow cytometry results. (a) CD4 + CD25 + T cells in serum. (b) OX62 + DCs in the spleen.

Compared with those in the sham group, the proportions of CD4 + CD25 + T cells in the remaining groups were significantly increased (*P* < 0.001; Figure 1a, Figure A1a). Compared with that in the model group, the proportion in the Tα1 group, VitC group, and Tα1 + VitC group was significantly decreased at 12, 24, and 48 h (*P* < 0.001), with the most noticeable decrease observed in the Tα1 + VitC group.

Flow cytometry of the spleen samples showed consistent outcomes (Figure 1b, Figure A1b). The proportion of OX62 + DCs in the model group, Tα1 group, VitC group, and Tα1 + VitC group was significantly increased compared with that in the sham group (*P* < 0.001). Compared with that in the model group, the proportion of OX62 + DCs in the Tα1 group, VitC group, and Tα1 + VitC group was significantly decreased at 12, 24, and 48 h (*P* < 0.001). Compared with that in the Tα1 group, the proportion of OX62 + DCs in the Tα1 + VitC group was significantly decreased (*P* < 0.05).

### 3.2 ELISA

#### 3.2.1 Blood samples

**Figure 2:**
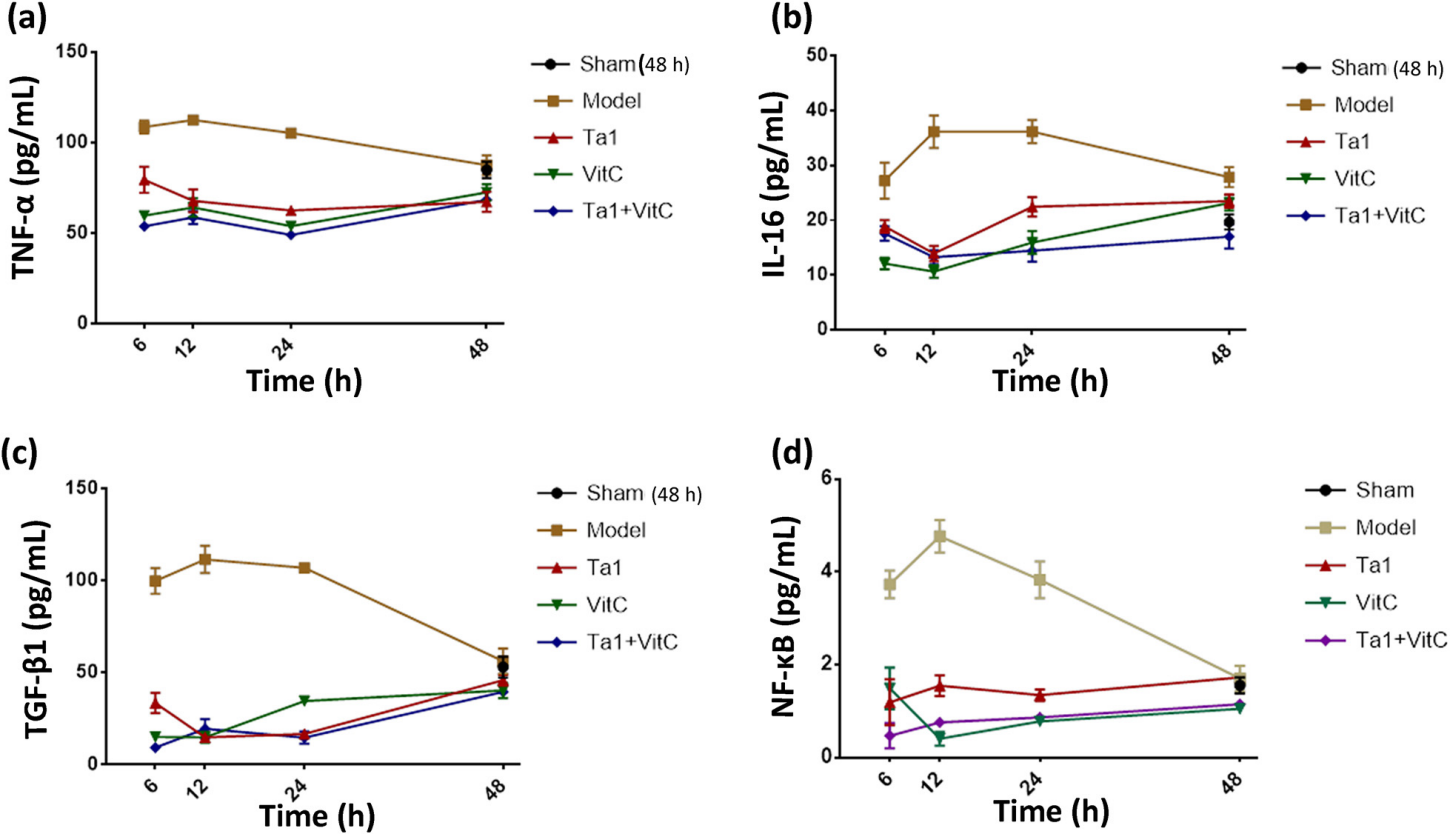
Comparison of the levels of serum cytokines among different groups according to ELISA. (a) TNF-α, (b) IL-6, (c) TGF-β1, and (d) NF-κB (P65).

Compared with that in the sham group, the serum level of TNF-α in the model group was significantly increased at 6, 12, and 24 h (*P* < 0.001, *P* < 0.01, and *P* < 0.05, respectively). Compared with that in the model group, the expression level of TNF-α in the Tα1 group, VitC group, and Tα1 + VitC group was significantly decreased at 6, 12, and 24 h (*P* < 0.05, *P* < 0.01, and *P* < 0.01, respectively) (Figure 2a).

Compared with that in the sham group, the expression level of IL-6 was significantly upregulated in the model group at 12, 24, and 48 h (*P* < 0.01). At 6 h, compared with that in the model group, the expression level of IL-6 was significantly downregulated in both the VitC group and the Tα1 + VitC group (*P* < 0.01 and *P* < 0.05, respectively). At 12 h, compared with that in the model group, the expression level of IL-6 was significantly downregulated in the Tα1, VitC, and Tα1 + VitC groups (*P* < 0.01, *P* < 0.001). At 48 h, compared with that in the model group, the expression level of IL-6 in the Tα1 + VitC group was significantly downregulated (*P* < 0.05) (Figure 2b).

Compared with that in the sham group, the TGF-β1 expression level was significantly upregulated in the model group at 6, 12, and 24 h (*P* < 0.001). Compared with that in the model group, TGF-1 expression was significantly downregulated in the Tα1, VitC, and Tα1 + VitC groups at these time points (*P* < 0.001). Compared with that in the model group, the TGF-β1 expression level was downregulated in the Tα1, VitC, and Tα1 + VitC groups, but there was no significant difference at 48 h (Figure 2c).

Compared with that in the sham group, NF-κB expression was significantly upregulated in the model group at 6, 12, and 24 h (*P* < 0.001). At these time points, NF-κB expression levels were significantly downregulated in all treatment groups compared with the model group (*P* < 0.01, *P* < 0.001). At 48 h, a significant decrease was still observed in the VitC group and Tα1 + VitC group (*P* < 0.05) (Figure 2d).

#### 3.2.2 Spleen samples

**Figure 3:**
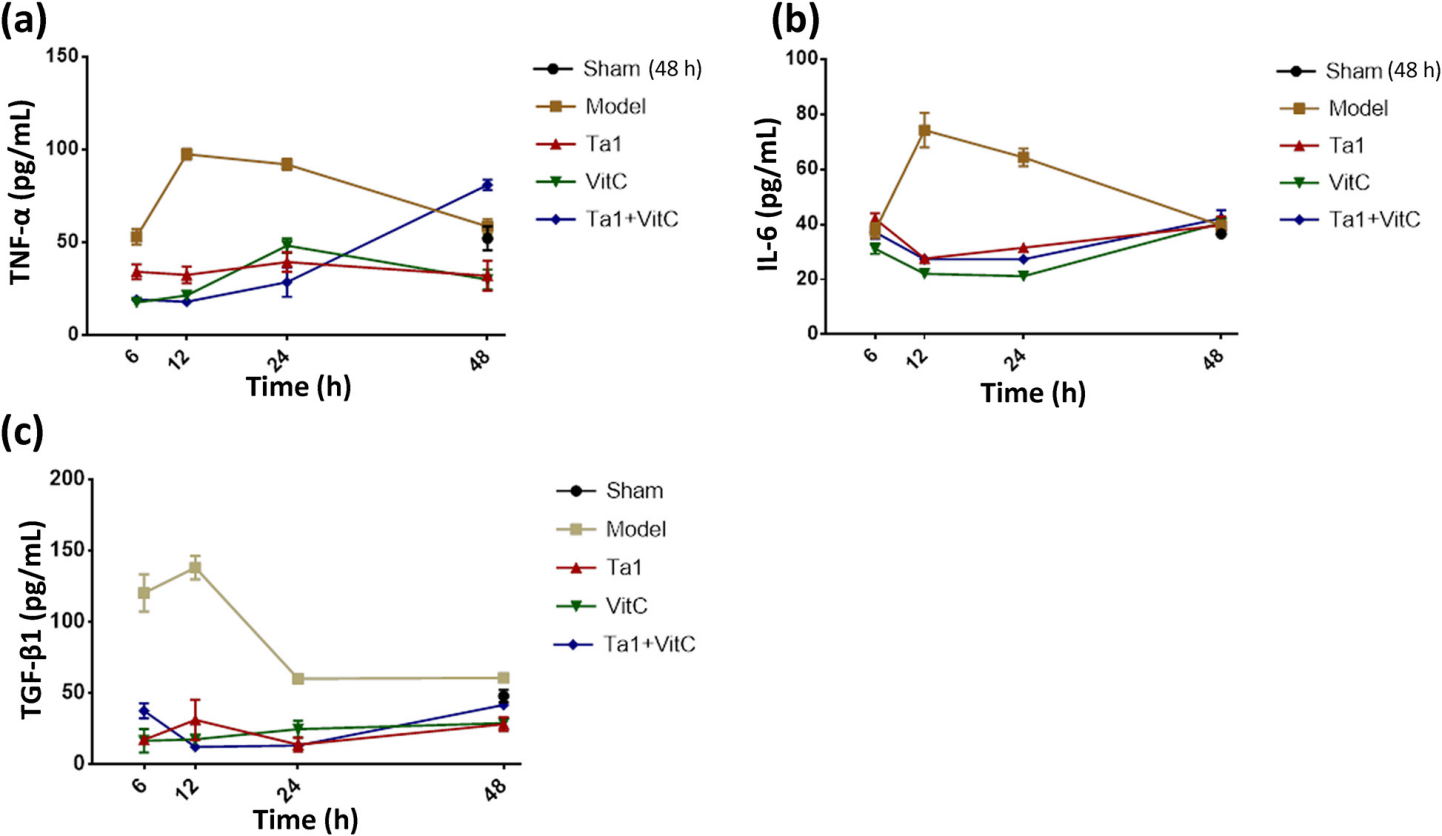
Comparison of the levels of spleen cytokines among different groups. (a) TNF-α, (b) IL-6, and (c) TGF-β1.

In spleen samples, compared with that in the sham group, the expression level of TNF-α in the model group was significantly upregulated at 12, 24, and 48 h (*P* < 0.01). Compared with that in the model group, the expression level of TNF-α was significantly downregulated in the Tα1 group, VitC group, and Tα1 + VitC group at 6, 12, and 24 h, with the most noticeable change observed in the combined group (*P* < 0.001). Compared with that in the model group, at 48 h, the expression level of TNF-α was downregulated in the VitC group, and the difference was statistically significant (*P* < 0.01). However, the expression level of TNF-α was upregulated in the Tα1 + VitC group, and the difference was statistically significant (*P* < 0.001). Combining the results of TNF-α in serum and the results of the Tα1 group and VitC group, we consider that the increase may have been caused by an experimental error (Figure 3a).

After modeling, the expression level of IL-6 increased in the model group at 12 and 24 h (*P* < 0.01 and *P* < 0.001, respectively). At these time points, all treatment groups exhibited significantly increased IL-6 levels compared with the model group (*P* < 0.001) (Figure 3b).

The TGF-β1 expression level also significantly increased at 6, 12, and 24 h after modeling. Compared with that in the model group, the TGF-β1 expression level was significantly downregulated in all treatment groups at 6, 12, 24, and 48 h (*P* < 0.01, *P* < 0.001) (Figure 3c).

### 3.3 HE staining

**Figure 4:**
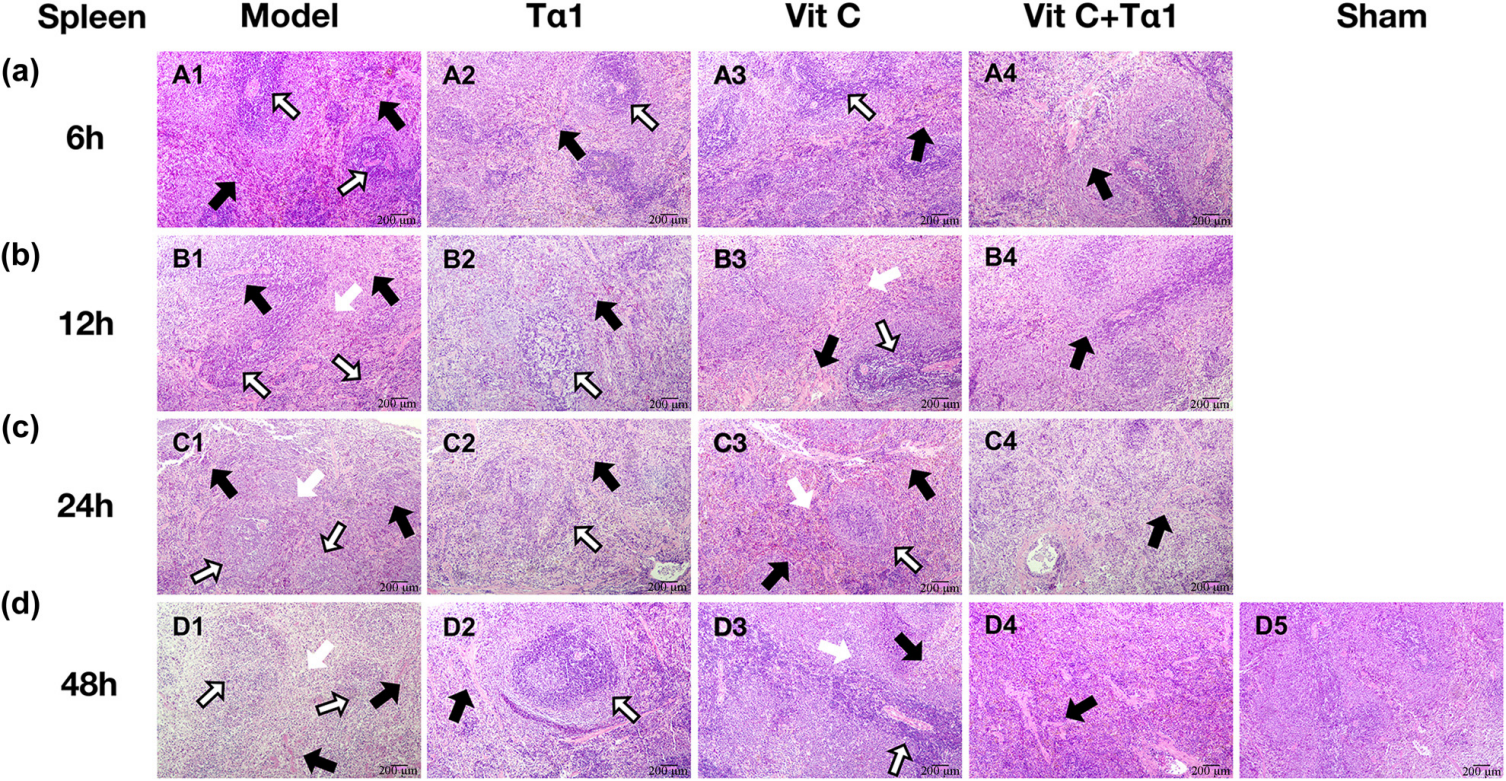
HE staining of the spleen tissue. (a) HE staining of spleen tissue in the 6 h subgroup (×200) (A1: Model group at 6 h; A2: Tα1 group at 6 h; A3: VitC group at 6 h; A4: Tα1 + VitC group at 6 h), (b) HE staining of spleen tissue in the 12 h subgroup (×200) (B1: Model group at 12 h; B2: Tα1 group at 12 h; B3: VitC group at 12 h; B4: Tα1 + VitC group at 12 h), (c) HE staining of spleen tissue in the 24 h subgroup (×100) (C1: Model group at 24 h; C2: Tα1 group at 24 h; C3: VitC group at 24 h; C4: Tα1 + VitC group at 24 h), and (d) HE staining of spleen tissue in the 48 h subgroup (×100) (D1: Model group at 48 h; D2: Tα1 at 48 h; D3: VitC group at 48 h; D4: Tα1 + VitC group at 48 h; D5: Sham group at 48 h). Reactive hyperplasia of the white pulp is indicated by a black hollow arrow, infiltration of inflammatory cells between red pulp by a black solid arrow, and interstitial fibrous tissue hyperplasia by a white arrow.

The results of HE staining are shown in Figure 4.

No abnormal changes were observed in the spleen tissue of the sham group (Figure 4D5). Unlike those in the sham group, the rats in the model group showed reactive hyperplasia of the white pulp (indicated by the black hollow arrow in Figure 4A1) and infiltration of inflammatory cells within the red pulp (indicated by the black solid arrow in Figure 4A1) in spleen tissue 6 h after modeling. Interstitial fibrous tissue hyperplasia appeared (indicated by the white arrow in Figure 4B1–D1, respectively) at 12, 24, and 48 h due to the white and red pulp changes.

In the Tα1 group, white pulp hyperplasia and intermedullary inflammatory cell infiltration were observed in the spleen tissue at 6, 12, 24, and 48 h (indicated by the black hollow and solid arrows in Figure 4A2–D2, respectively). In the VitC group, white pulp hyperplasia and intermedullary inflammatory cell infiltration were observed at 6 h (indicated by the black hollow and solid arrow in Figure 4A3), and interstitial fibrous tissue hyperplasia appeared after 12 h (indicated by the white arrow in Figure 4B3–D3).

The rats in the Tα1 + VitC group only showed infiltration of inflammatory cells in the red pulp in spleen tissue at 6–48 h (indicated by the black solid arrow in Figure 4A4–D4). This group exhibited no white pulp hyperplasia or interstitial fibrous tissue hyperplasia, and the infiltration of inflammatory cells in the red pulp was slight.

## 4 Discussion

Tregs express CD4 and CD25. CD4 + CD25 + T cells are a recently recognized class of immunomodulatory cells that originate in the thymus and exert suppressive immunomodulatory effects. In this study, at 6, 12, 24, and 48 h after modeling, the proportion of CD4 + CD25 + T cells in the Tα1, VitC, and Tα1 + VitC groups was significantly reduced compared with that in the model group, and we also found that the proportion in the Tα1 + VitC group was more significantly reduced than that in the Tα1 group and the VitC group. According to the literature [23], Treg numbers and TNF-α levels in the peripheral blood of septic rats are decreased by Tα1 compared with the CLP group after 3 days of modeling. Our finding in the Tα1 group was consistent with that reported in the literature. A subset of CD4 + CD25 + T cells expresses CD45RO isoforms, GITC, and CD152 on the cell surface and the transcription factor Foxp3 in the nucleus. There is growing evidence that CD4 + CD25 + Foxp3 + Tregs play an active role in reducing sepsis-induced inflammation through TGF-β-dependent and TGF-β-independent pathways [24,25,26,27,28]. Presumably, Tα1 and/or VitC reduce the expression of CD4 + CD25 + T cells and reduce the proliferation of Treg cells that inhibit T cells, thereby mitigating the early stage of sepsis-induced rapid inflammation due to an excessive inflammatory response and the associated damage to the body.

DCs are named for the many dendritic or pseudopod-like protrusions that extend at maturity and are the most powerful antigen-presenting cells observed to date. The only function of DCs is antigen presentation. Our results showed that at all observed time points after modeling, the proportion of OX62 + DCs in the Tα1, VitC, and Tα1 + VitC groups was significantly reduced compared with that in the model group, and the proportion in the Tα1 + VitC group was more significantly reduced than that in the Tα1 and VitC groups. Despite the effectiveness of Tα1 + VitC, our findings have not been previously reported. We hypothesized that the combination of Tα1 and VitC reduced the levels of Tregs and DCs, thereby reducing the activation of NF-κB and the expression level of inflammatory factors.

In the course of sepsis, DCs first transfer to the antigen epitopes of neutrophils, macrophages, and T helper lymphocytes (Th), activate NF-κB, enter the nucleus and form a complex with DNA, induce cell apoptosis, and release numerous proinflammatory cytokines and chemokines, such as TNF-α and IL-6. Therefore, inflammatory factors serve as indicators of the degree of the inflammatory response and can also be used as indicators of sepsis patient prognosis. Numerous studies have shown that during sepsis, the massive release of cytokines and the dysregulation of the inflammatory response due to tissue damage are among the origins of the pathophysiology leading to vascular endothelial damage [29,30]. According to a clinical study involving 81 patients with severe pneumonia complicated with sepsis, the expression levels of TNF-α, IL-6, IL-8, and other inflammatory factors in the group that received Tα1 under the basal epithelium were significantly reduced compared with those in the control group [31]. The efficacy of VitC in the treatment of sepsis has also been reported in numerous studies [32,33]. In this study, the Tα1 and VitC groups both exhibited reduced relevant inflammatory factors, consistent with the abovementioned results. The antisepsis effect of VitC is possibly realized by suppressing immune dysfunction and inflammation-associated functional processes [34].

The spleen is the largest lymphoid organ in the human body, with the functions of blood storage, hematopoiesis, removal of aging red blood cells, and immune responses. The spleen comprises three parts, the white pulp, red pulp, and marginal area. The white pulp is composed of dense lymphocytes and is the main location of specific immunity. Our experimental results showed no abnormal changes in the spleen tissue of rats in the sham group. At 6–48 h after modeling, compared with the sham group, the spleen tissue of the rats in the model group showed pathological changes, such as white myeloid reactive hyperplasia, fibrous tissue hyperplasia, and infiltration of red medullitis cells. Compared with the model group, the spleen tissue of rats in the Tα1 and VitC groups still demonstrated white myeloid reactive hyperplasia, but the infiltration of red medullitis cells decreased. Spleen tissue infiltration in rats in the Tα1 + VitC group showed red medullitis cell infiltration, but there was no interstitial fibrous tissue hyperplasia, and no pathological changes were seen in the white pulp. These results suggest that the combination of Tα1 + VitC has a therapeutic effect on septic rats and can effectively reduce the organ damage caused by sepsis and regulate the body’s immune response. Simultaneous intergroup comparisons suggest that Tα1 or VitC alone may not be as effective as Tα1 + VitC combination therapy.

The mechanism of action of sepsis is complex, including an excessive inflammatory response, immunosuppression, a second strike, apoptosis, oxidative stress and coagulation disorders, which are jointly involved in organ damage. Carlson et al. [35] reported that the combined use of VitC and VitE in septic rats prevented the activation of NF-κB and caspase and improved myocardial contractile function, suggesting that the combined use of these antioxidants has an important protective effect on sepsis-related myocardial dysfunction. In contrast, a high dose of VitC (1,000 m/8 h) combined with α-tocopherol (1,000 IU/8 h) significantly reduced the duration of mechanical ventilation in mechanically ventilated patients [36]. In addition, the combination of VitC and VitE significantly reduced the levels of oxidative stress and lipid peroxidation, downregulated the expression of hepatic cytochrome p450 enzymes in sepsis and prevented the dysfunction of hepatic drug metabolism in sepsis [37]. These results suggest that the combined use of VitC and VitE can exert a better antioxidant effect than individual treatment, which is clearly beneficial for improving the patient’s condition and prognosis. The use of Tα1 in the early stages of sepsis regulates immune function, attenuates overreaction, prevents immunosuppression, and avoids or mitigates secondary strikes, while the use of VitC, which inhibits the expression of superoxides in microvascular endothelial cells, reduces endothelial oxidative stress and improves endothelial dysfunction and microcirculatory disorders [38]. Furthermore, VitC can inhibit the expression of TNF-α-induced intracellular adhesion molecule 1, which mediates the production of oxidative stress via reactive oxygen species and nitric oxide synthase (NOS) [39], preventing platelet and leukocyte adhesion to the microvascular endothelium and improving microvascular blood flow. In addition, VitC prevents endothelial-type nitric oxide synthase (eNOS) production, reduces protein phosphatase 2A activity, inhibits vascular leakage, and attenuates vascular endothelial barrier dysfunction by preventing eNOS uncoupling (superoxide anion production), reducing inducible nitric oxide synthase, and neural-type nitric oxide synthase activity and endothelial permeability [40], and reversing vascular hyporesponsiveness and hypotension in patients with sepsis [41].

Tα1 is produced by thymic epithelial cells and thymic endocrine cells and is an endogenous regulator of the innate and acquired immune systems [42]. Tα1 mainly enhances T-cell function, in addition to acting through different toll-like receptors (type I transmembrane proteins, TLRs) on different DC subpopulations and myeloid differentiation protein antigen (MyD88)-dependent signaling pathways, thus playing a unique role in balancing pro- and anti-inflammatory cytokines. The therapeutic effects and mechanisms of thymidine α1 in chronic viral hepatitis, malignant tumors, and acquired immune deficiency syndrome (AIDS) have been clearly established, while the therapeutic effects in severe infections, especially sepsis, have been confirmed in several clinical studies. However, the mechanisms of action are still not well understood. The most commonly used immunomodulatory drugs in clinical treatments are Tα1 and ustekin, and the results of one study showed that immunotherapy reduced the incidence of 28 days death in patients with severe sepsis but had no effect on the length of ICU stay, duration of mechanical ventilation, or use of vasoactive drugs and antibiotics [43]. Tα1 significantly increased the expression of serum monocyte human leukocyte antigen (mHLA)-DR in patients with severe sepsis, indicating that this treatment may help improve the clinical prognosis of patients with severe sepsis.

VitC, also known as ascorbic acid, is a water-soluble antioxidant and cofactor for some enzymes [44] and is an important cofactor for iron- and copper-containing enzymes [45]. VitC is absorbed in the small intestine via sodium-dependent VitC transporters and excreted via the kidneys [46]. Plasma VitC levels are significantly lower in patients with sepsis, and plasma VitC levels are 1/3 to 1/2 of the normal level in patients with severe sepsis [47,48,49]. In a study of the acute inflammatory response induced by intravenous administration of low-dose *Escherichia coli* endotoxin (LPS) in healthy subjects, a 37% decrease in plasma VitC concentrations was observed after 4 h [50], and reduced plasma VitC concentrations were significantly associated with inflammatory infection, the severity of organ failure, morbidity, and mortality [51]. VitC is also a potent antioxidant and cofactor of biosynthetic and gene regulatory enzymes [52], and severe VitC deficiency can lead to the potentially fatal disease scurvy [53]. Scurvy is characterized by a weakened collagen structure leading to poor wound healing and impaired immune function. Patients with scurvy are highly susceptible to fatal infectious diseases, such as pneumonia, and conversely, infections significantly affect VitC levels due to enhanced inflammation and metabolic demands [54]. Recent studies have shown the beneficial effects of VitC on multiple pathways associated with sepsis. VitC deficiency has been shown to worsen pulmonary pathology following influenza infection. In addition, VitC levels are significantly reduced in critically ill patients, especially those with proinflammatory diseases (e.g., sepsis and SIRS) [55]. In addition, there is a dysregulated host immune response in sepsis, and immunosuppression can occur early in the course of the disease [56]. The immunosuppressed state and the imbalance in the pro- and anti-inflammatory responses can lead to an increased mortality rate [57]. VitC is an important immune response factor that can improve host defenses by regulating the proliferation and differentiation of immune cells, regulating the balance of helper T lymphocyte 1/helper T lymphocyte 2, and improving the functions of macrophages, T lymphocytes, and B lymphocytes. In the present study, the inflammatory response to sepsis in rats was differentially reduced and immune function was differentially modulated after Tα1 and/or VitC intervention, and these outcomes were accompanied by reductions in the proportions of CD4 + CD25 + T cells and DCs and reductions in the inflammatory cytokines IL-6, TNF-α, TGF-β1, and NF-κB (P65).

The accumulation of proinflammatory factors in patients is associated with early morbidity and mortality, and the number of chronic sepsis-related deaths appears significantly associated with immunosuppression and dysfunction; most sepsis-related deaths occur after 3 days, and some occur several weeks after the onset of disease [10]. Our findings suggest that Tα1, VitC, and Tα1 + VitC treatment in the early proinflammatory phase of sepsis may improve immune function in rats with sepsis, attenuating the overly dysregulated inflammatory response, reducing inflammatory damage, and protecting organ function. The effective treatment effect was particularly reflected in the Tα1 + VitC group, in which no noticeable white pulp hyperplasia or interstitial fibrous tissue hyperplasia was observed at different time points. In patients with sepsis, in addition to traditional active anti-infection and proper shock treatment, combined Tα1 and VitC treatment may help modulate the harmful immune proinflammatory state in the early stages. However, the synergistic action between Tα1 and VitC remains to be explored.

There are some limitations in the present study. First, animal experimental studies are sometimes incomplete, and the extrapolation of results obtained through animal experimental models to other species is sometimes unreliable. Second, animal experiments are sometimes misleading, and many of the apparent abnormalities observed between animal tests and humans may be due to normal biological characteristic responses that are specific to the species or changes caused by unnatural means or stress reactions in the laboratory environment. These abnormalities are unrelated to pathological changes in humans. In our study, the small sample size was a major experimental limitation, and the number of blank controls was too small to allow for blank comparisons at each time point.

## 5 Conclusion

Our results suggest that Tα1 + VitC treatment can improve immune function, reduce inflammatory damage, and protect organ function in septic rats. In patients with sepsis, in addition to traditional active anti-infection and proper shock treatment, the combination of Tα1 and VitC can be considered a treatment and may help regulate the immune state and improve prognosis.
